# Mutational effects of human dopamine transporter at tyrosine88, lysine92, and histidine547 on basal and HIV-1 Tat-inhibited dopamine transport

**DOI:** 10.1038/s41598-019-39872-1

**Published:** 2019-03-07

**Authors:** Wei-Lun Sun, Pamela M. Quizon, Yaxia Yuan, Matthew J. Strauss, Richard McCain, Chang-Guo Zhan, Jun Zhu

**Affiliations:** 1 Department of Drug Discovery and Biomedical Sciences, College of Pharmacy, University of South Carolina, Columbia, SC USA; 2 Molecular Modeling and Biopharmaceutical Center, University of Kentucky, Lexington, KY USA; 3 Department of Pharmaceutical Sciences, College of Pharmacy, University of Kentucky, Lexington, KY USA

## Abstract

Dysregulation of dopaminergic system induced by HIV-1 Tat protein-mediated direct inhibition of the dopamine transporter (DAT) has been implicated as a mediating factor of HIV-1 associated neurocognitive disorders. We have reported that single point mutations on human DAT (hDAT) at tyrosine88 (Y88F), lysine92 (K92M), and histidine547 (H547A) differentially regulate basal dopamine uptake but diminish Tat-induced inhibition of dopamine uptake by changing dopamine transport process. This study evaluated the effects of double (Y88F/H547A) and triple (Y88F/K92M/H547A) mutations on basal dopamine uptake, Tat-induced inhibition of DAT function, and dynamic transport process. Compared to wild-type hDAT, the V_max_ values of [^3^H]Dopamine uptake were increased by 96% in Y88F/H547A but decreased by 97% in Y88F/K92M/H547A. [^3^H]WIN35,428 binding sites were not altered in Y88F/H547A but decreased in Y88F/K92M/H547A. Y88F/H547A mutant attenuated Tat-induced inhibition of dopamine uptake observed in wild-type hDAT. Y88F/H547A displayed an attenuation of zinc-augmented [^3^H]WIN35,428 binding, increased basal dopamine efflux, and reduced amphetamine-induced dopamine efflux, indicating this mutant alters transporter conformational transitions. These findings further demonstrate that both tyrosine88 and histidine547 on hDAT play a key role in stabilizing basal dopamine transport and Tat-DAT integration. This study provides mechanistic insights into developing small molecules to block multiple sites in DAT for Tat binding.

## Introduction

About thirty-seven million people are currently living with HIV-1 infection worldwide, leading to a significant global public health problem. While the effective antiretroviral therapies significantly reduced the mortality rate in the patients with HIV-1 infection, nearly 50% of HIV-1 infected patients have various degrees of neurological complications that are referred to as HIV-1-associated neurocognitive disorders (HAND)^1^. The continuous exposure of the central nerves system to HIV-1 viral proteins, inflammation, and antiretroviral agents results in neuropathological and neurocognitive deficits observed in the patients with HAND^2–7^. Transactivator of transcription (Tat) protein, one of seven HIV-1 viral proteins, has been shown to play a critical role in HIV-1 viral replication as well as the development of HAND^2,8^, which can be exacerbated by concurrent cocaine abuse^9^. Thus, developing an intervention strategy in the early stage of HIV-1 infection would prevent the development of neurocognitive dysfunction in HIV-1 infected individuals.

Normal dopaminergic transmission is important for maintaining different brain activities including attention, learning, memory^10,11^, and motivation^12,13^. Dopamine (DA) transporter (DAT) is a presynaptic membrane protein that reuptakes the released DA from the synaptic cleft back into cytosol, maintaining a stable DA homeostasis. The DAT activity is directly inhibited by HIV-1 Tat protein and cocaine, which synergistically enhances synaptic DA levels^9^. The dysregulation of DA system is a mediating factor of HAND as well as a factor in cocaine abuse^14,15^. Using computational modeling and experimental approach, we have identified several key residues on human DAT (hDAT), which are crucial for Tat-hDAT interaction and dynamic transport process^9^. Furthermore, we have demonstrated that *in vitro* exposure to Tat reduced reuptake of DA via hDAT in cells^16–18^ and rat striatal synaptosomes^19^. The inhibitory effect of Tat on DAT function results from Tat directly interacting with DAT^16,20,21^. Single point mutations of hDAT at tyrosine88 (to phenylalanine, Y88F), lysine 92 (to methionine, K92M), histidine547 (to alanine, H547A) differentially alter basal DA uptake but attenuate the Tat inhibitory effects on DA transport^17,18^. For example, DA uptake is decreased in K92M and increased in H547A, respectively; however Y88F mutant preserves basal DA uptake^17,18^. Notably, the mutational effects on normal DA uptake and Tat inhibitory effect on DAT function are associated with alterations of transporter conformational transitions^9^.

We have demonstrated that Tat protein inhibits DAT function in an allosteric modulatory mechanism^19,22^. Recent studies have demonstrated that novel SRI-compounds exhibit a partial antagonistic role in DAT function as allosteric modulators^23–25^. We have reported that SRI-30827, one of the novel allosteric modulators, blocks Tat interaction with DAT^26^. Thus, identifying the specific binding sites on hDAT for Tat and its role in DA transport process could be beneficial to attenuation of the inhibitory effect of Tat on DAT-mediated dopaminergic transmission. On the other hand, through an allosteric modulatory mechanism, the inhibitory effect of Tat on DAT function can also be diminished by targeting the specific DAT residues that are distinct from Tat binding sites. However, based on our computational structural models for Tat binding with hDAT, the interaction of Tat with hDAT involves multiple residues of DAT^9,21^ and our previous results obtained from single point mutations of DAT only present the role of a particular residue in Tat-DAT interaction. Therefore, this study investigated the mutational effects of Y88F/H547A and Y88F/K92M/H547A on basal DAT function, Tat inhibitory effect on DA uptake, and dynamic DA transport process.

## Results

### Computational modeling: Impact of Tyr88/His547 and Tyr88/Lys92/His547 on functional relevance of human DAT

**Figure 1:**
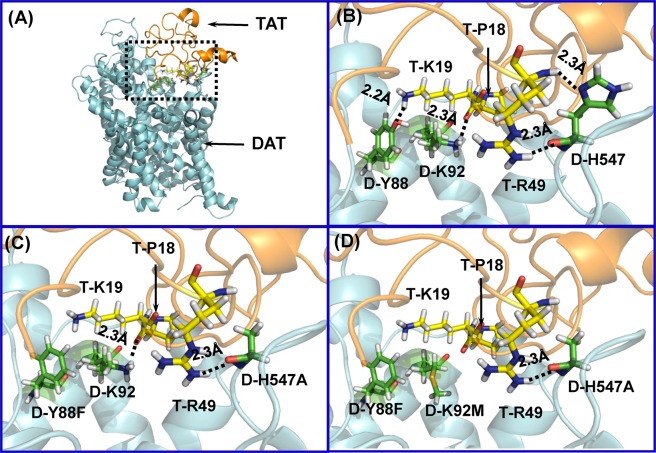
Key residues, D-Y88, D-K92 and D-H547 involved in the HIV-1 Tat-DAT binding. (**A**) Typical Tat-DAT binding complex from MD trajectory. Tat and DAT are represented as gold and cyan ribbons, respectively. The dashed box indicates the binding surface between Tat and DAT. (**B**) Tat residues, T-P18, T-K19 and T-R49, are represented as ball-stick style and colored in yellow. DAT residues, D-Y88, D-K92 and D-H547, are represented as ball-stick style and colored in green. Dashed lines represent inter-molecular hydrogen bonds with labeled distances. (The prefix T- and D- indicates residues of Tat and DAT, respectively). (**C**) Double mutation D-Y88F/D-H547A on hDAT-TAT structure. D-H547A mutation eliminates one hydrogen bond with T-R49, and D-Y88F mutation eliminates the hydrogen bond with T-K19. (**D**) Triple mutation D-Y88F/D-K92M/D-H547A on hDAT-TAT structure. Addition of a triple mutation of D-Y88F, D-K92M, and D-H547A, also eliminates the hydrogen bond with T-P18.

Based on the constructed hDAT-Tat binding model in our previous work^20^, hDAT residues Y88, K92, and H547 could form hydrogen bonds with residues K19, P18, and R49 of HIV-1 Tat, respectively. According to our previous computational and experimental results, single mutation of either hDAT Y88 (Y88F), K92 (K92M) or H547 (H547A) could significantly attenuate the binding between DAT and Tat^17,18^. As shown in Fig. 1A,B, hDAT Y88, K92 and H547 residues independently interact with Tat, suggesting an effect for double or triple mutation on disturbing DAT-Tat binding. For example, two hydrogen bonds in the DAT-Tat interface were eliminated by double mutation of Y88F/H547A on hDAT (Fig. 1C), and three hydrogen bonds in the DAT-Tat interface were eliminated by triple mutation of Y88F/K92M/H547A on hDAT (Fig. 1D). Therefore, one may expect that Y88F/H547A and Y88F/K92M/H547A mutants are more effective in inhibiting DAT-Tat binding than single point mutations of Y88F, K92M and H547A.

### Mutations of Y88/H547 and Y88/K92/H547 differentially alter DA uptake kinetics and DAT binding

**Figure 2:**
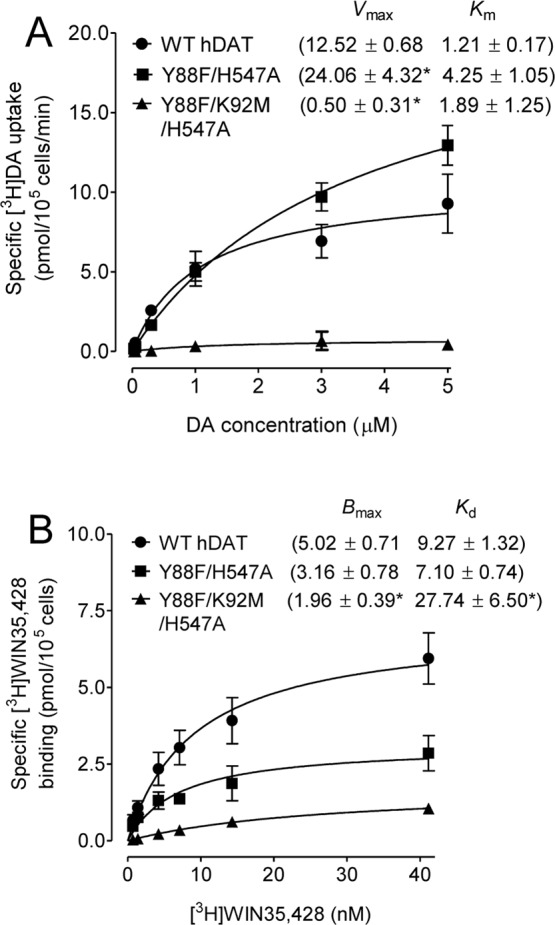
Effects of Y88F/H547A and Y88F/K92M/H547A on [^3^H]DA uptake and [^3^H]WIN35,428 binding. (**A**) Kinetic parameters (V_max_ and K_m_) of [^3^H]DA uptake in WT hDAT and mutants. [^3^H]DA uptake assay was conducted in PC12 cells transfected with WT hDAT and mutants by incubation with one of 6 mixed concentrations of the [^3^H]DA as the total rate of DA uptake. Nonspecific uptake of [^3^H]DA was determined by a parallel incubation of each mixed DA concentrations with nomifensine (10 µM, final concentration). Specific [^3^H]DA uptake (V_max_) was calculated by subtraction of nonspecific DA uptake from total DA uptake. The parameter values were presented as means ± S.E.M. *p < 0.05 compared to WT hDAT value (unpaired Student’s *t* test) (n = 4–5/group). (**B**) Saturation analysis of [^3^H]WIN35,428 binding in WT hDAT and mutants. *B*_max_ and *K*_d_ values were estimated by fitting one-site binding and presented as means ± S.E.M. *p < 0.05 compared to WT hDAT value (unpaired Student’s *t* test) (n = 4–12/group).

We have demonstrated that single point mutations on hDAT at His547 (H547A) and Lys92 (K92M) displayed a 196% increase and a 71% decrease in the V_max_ of [^3^H]DA uptake, respectively, while Y88F preserved normal DA uptake^17,18^. The current study determined the multiple mutations of these residues on DA uptake and DAT binding sites. As shown in Table 1 and Fig. 2A, compared to WT hDAT (12.52 ± 0.68 pmol/min/10^5^ cells), the V_max_ values were increased by 92% in Y88F/H547A (24.06 ± 4.32 pmol/min/10^5^ cells, *t*_(7)_ = 2.33, *p* < 0.05) and dramatically decreased in Y88F/H547A/K92M (0.5 ± 0.31 pmol/min/10^5^ cells, *t*_(6)_ = 16.2, *p* < 0.001), respectively. Additionally, compared to WT hDAT (1.21 ± 0.17 µM), the K_m_ value in Y88F/H547A was increased by 250% (4.25 ± 1.05 µM, *t*_(7)_ = 2.54, *p* < 0.05) but not altered in Y88F/K92F/H547A.

**Table 1:** Kinetic properties and inhibitory activities in [^3^H]DA uptake in WT and mutated hDAT.

	*V*_max_ (pmol/min/10^5^ cells)	*K*_m_ (µM)	IC_50_ (nM)			
			DA	Cocaine	GBR12909	AMPH
WT hDAT	12.52 ± 0.68	1.21 ± 0.17	2063 ± 582.5	350 ± 49.4	511 ± 56.6	926 ± 167.4
Y88F/H547A	24.06 ± 4.32*	4.25 ± 1.05*	7918 ± 637.6*	131 ± 13.9*	253 ± 40.4*	1279 ± 200.5
Y88F/ K92M/H547A	0.5 ± 0.31*	1.89 ± 1.25	ND	ND	ND	ND

Data are presented as mean ± S.E.M. values from four to six independent experiments performed in duplicates. *p < 0.05 compared with WT hDAT (unpaired Student’s *t* test). ND. Not determined.

In the hDAT, the [^3^H]WIN35,428 binding sites share pharmacological identity with the DA uptake site and is also part of the cocaine binding domain^27,28^. Kinetic analysis of [^3^H]WIN35,428 binding was conducted to determine the effects of multiple mutations of hDAT on the DA binding site. As shown in Table 2 and Fig. 2B, in comparison to WT hDAT, neither B_max_ nor K_d_ values of [^3^H]WIN35,428 were altered in Y88F/H547A, whereas Y88F/K92M/H547A displayed a 61% decrease in the B_max_ value (*t*_(14)_ = 2.4, *p* < 0.05) and a 199% increase in the K_d_ value (*t*_(14)_ = 4.4, *p* < 0.001). Thus, due to the significant reduction on DA uptake for Y88F/K92M/H547A, only the Y88F/H547A mutant was included for the rest of experiments.

**Table 2:** Kinetic properties and inhibitory activities in [^3^H]WIN 35,428 binding in WT and mutated hDAT.

	B_max_(pmol/10^5^ cells)	K_d_ (nM)	IC_50_ (nM)		
			DA	Cocaine	GBR12909
WT hDAT	5.02 ± 0.71	9.27 ± 1.32	2291 ± 487.3	235 ± 53.3	485 ± 49.4
Y88F/H547A	3.16 ± 0.78	7.10 ± 0.74	7236 ± 1825*	1494 ± 333.0*	452 ± 113.0
Y88F/K92M/H547A	1.96 ± 0.39*	27.74 ± 6.50*	ND	ND	ND

Data are presented as mean ± S.E.M. of IC_50_ values from 4–12 independent experiments performed in duplicates. ^*^p < 0.05 compared with WT hDAT (unpaired student’s *t* test). ND. Not determined.

### Mutation of Y88/H547 alters the inhibition potency of DA uptake and DAT binding by substrates and inhibitors

To characterize the influence of Y88F/H547A on DA transport and the specific binding site in hDAT for DA, cocaine, GBR12909, and amphetamine (AMPH), we determined the ability of these DAT inhibitors or substrate to inhibit [^3^H] DA uptake in WT hDAT and Y88F/H547A (Table 1). In comparison to WT hDAT, Y88F/H547A reduced the apparent affinity (IC_50_) for DA [WT hDAT: 2063 ± 582.5 nM; Y88F/H547A: 7918 ± 637.6 nM; *t*_(10)_ = 6.78, *p* < 0.0001]. In contrast, compared to WT hDAT, Y88F/ H547A displayed an increase in IC_50_ for cocaine [WT hDAT: 350 ± 49.4 nM; H547A/Y88F: 131 ± 13.9 nM; *t*_(6)_ = 4.28, *p* < 0.01] and GBR12909 [WT hDAT: 511 ± 56.6 nM; Y88F/H547A: 253 ± 40.4 nM; *t*_(8)_ = 3.71, *p* < 0.01]. We also evaluated the mutational effect of Y88F/H547A on the IC_50_ of DA, cocaine, and GBR12909 for inhibiting [^3^H]WIN35,428 binding (Table 2). Compared to WT hDAT, Y88F/H547A increased the IC_50_ values for DA (WT hDAT: 2291 ± 487.3 nM; Y88F/H547A: 7236 ± 1825 nM) and cocaine (WT hDAT: 235 ± 53.3; Y88F/H547A: 1494 ± 333.0 nM, *t*_(12)_ = 3.73, *p* < 0.001]. No difference in IC_50_ GBR12909 inhibiting [^3^H]WIN35,428 binding was observed between WT hDAT and Y88F/H547A.

### Effect of Y88F/H547A on basal PKC-mediated regulation of DAT function

**Figure 3:**
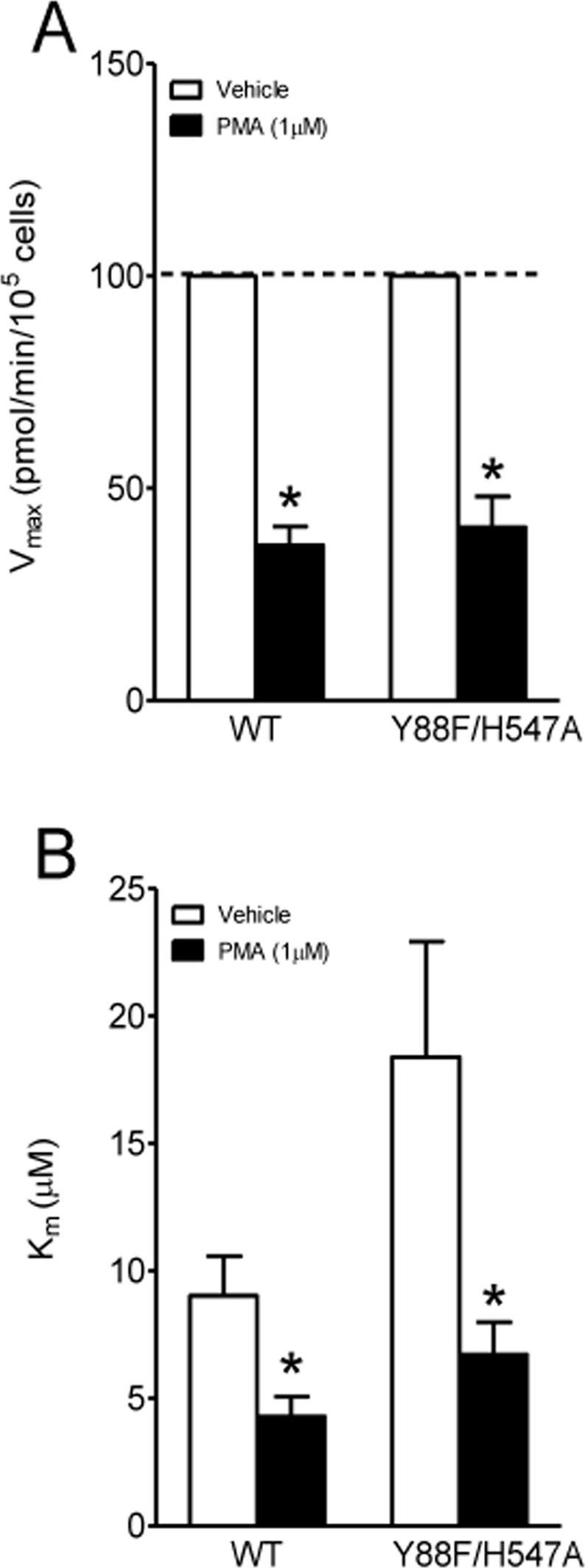
Effects of PMA on basal PKC-regulated DAT function in WT hDAT and Y88F/H547A mutant. Kinetic analysis of [^3^H]DA uptake was conducted in the presence or absence of PMA (PKC activator, 1 µM). (**A**) *V*_max_ and (**B**) *K*_m_. Data were presented as means ± S.E.M. (n = 6/group). *p < 0.05 compared to their respective controls (paired t-test after two-way ANOVA analyses).

The activation of PKC by phorbol 12-myristate 13-acetate (PMA), a PKC activator, decreases DA uptake through DAT^29,30^. We have demonstrated that the addition of 1 µM PMA produced a 40% and 60% decrease in the V_max_ of DA uptake in WT hDAT and H547A mutant, respectively, compared to the respective control, suggesting that H547A displays increased sensitivity to PMA-induced DAT phosphorylation^18^. In the current study, we also found that Y88F/H547A displayed increased the V_max_ relative to WT hDAT. We then determined whether Y88F/H547A-upregulated DA uptake is also mediated by alteration of sensitivity to PMA-induced DAT phosphorylation (Fig. 3). A two way ANOVA analysis on the V_max_ values of [^3^H]DA uptake revealed a significant main effect of PMA [F_(1, 20)_ = 9.40, *p* < 0.01]. No significant mutation × PMA treatment interaction [F_(1, 20)_ = 2.62; *p* = 0.076] was found. Addition of PMA reduced the V_max_ values in WT hDAT by 51% [*t*_(5)_ = 2.79, *p* < 0.05] and Y88F/H547A by 60% [*t*_(5)_ = 3.90, *p* < 0.05], respectively, compared to their respective vehicle controls. Similarly, two-way ANOVA analysis on the K_m_ values revealed significant main effects of mutation [F_(1, 20)_ = 5.52, *p* < 0.05] and PMA treatment [F_(1, 20)_ = 10.71, *p* < 0.001]. No significant interaction of mutation × PMA treatment was observed. The addition of 1 µM PMA reduced K_m_ values by 53% in WT hDAT [*t*_(5)_ = 3.31, *p* < 0.05] and 63% in Y88F/H547A [*t*_(5)_ = 2.54, *p* < 0.05], respectively. These results suggest that the double mutation of Tyr88 and His547 attenuates the single mutant H547A-increased sensitivity of PMA-induced decrease in DA uptake.

### Double Mutation of H547/Y88 attenuate Tat-induced inhibition of DAT

**Figure 4:**
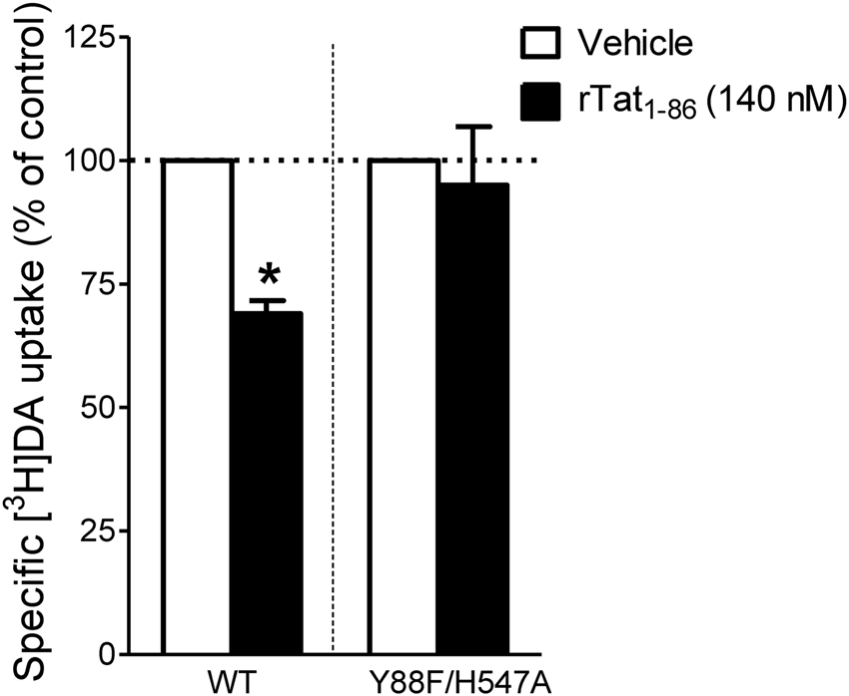
Effects of Tat on kinetic analysis of [^3^H]DA uptake. PC12 cells transfected with WT hDAT or Y88F/H547A were preincubated with vehicle or recombinant Tat_1–86_ (rTat_1–86_) at room temperature for 20 min followed by [^3^H]DA application. Data were calculated as mean ± S.E.M. of specific [^3^H]DA uptake relative to respective controls as 100% for individual experiment (WT hDAT: 1109.30 ± 230.27 dpm and Y88F/H547A: 400.13 ± 81.75 dpm and n = 5/group). *p < 0.05 compared to their respective controls (paired *t* test).

Our previous studies demonstrated that single point mutation of Tyr88 (Y88F) or His547 (H547A) attenuated Tat-induced inhibition of [^3^H]DA uptake observed in WT hDAT^17,18^. Since the triple mutant (Y88F/K92M/H547A) displayed an extremely low V_max_ of [^3^H]DA uptake, only the double mutant (Y88F/H547A) was tested for its effect on Tat-induced inhibition of DA uptake. Based on the computational modeling predictions (Fig. 1), the double mutation of Tyr88 and His547 would eliminate two pairs of hydrogen bonds between D-Y88-T-K19 and D-H547-T-R49 and impair Tat binding on hDAT, thereby attenuating Tat-induced inhibition of DA uptake. Due to the difference in the specific [^3^H]DA uptake between WT hDAT and Y88F/H547A mutants (Fig. 2), the inhibitory effects of Tat on DAT function in WT and Y88F/H547A were presented as % of Tat-mediated [^3^H]DA uptake relative to their respective controls (in the absence of Tat). As shown in Fig. 4, the application of 140 nM recombinant Tat_1–86_ (rTat_1–86_) significantly reduced the specific [^3^H] DA uptake in WT hDAT by 31% relative to its control [*t*_(4)_ = 11.54, *p* < 0.001]. However, the Tat-induced reduction of [^3^H] DA uptake in WT hDAT was attenuated in Y88F/H547A compared to its control [*t*_(4)_ = 0.42, *p* > 0.05], suggesting that the double mutation of Tyr88 and His547 attenuates Tat’s inhibitory effect on DA uptake.

### Effects of double mutation of Y88/H547 on zinc regulation of DAT conformational transitions and basal DA efflux

**Figure 5:**
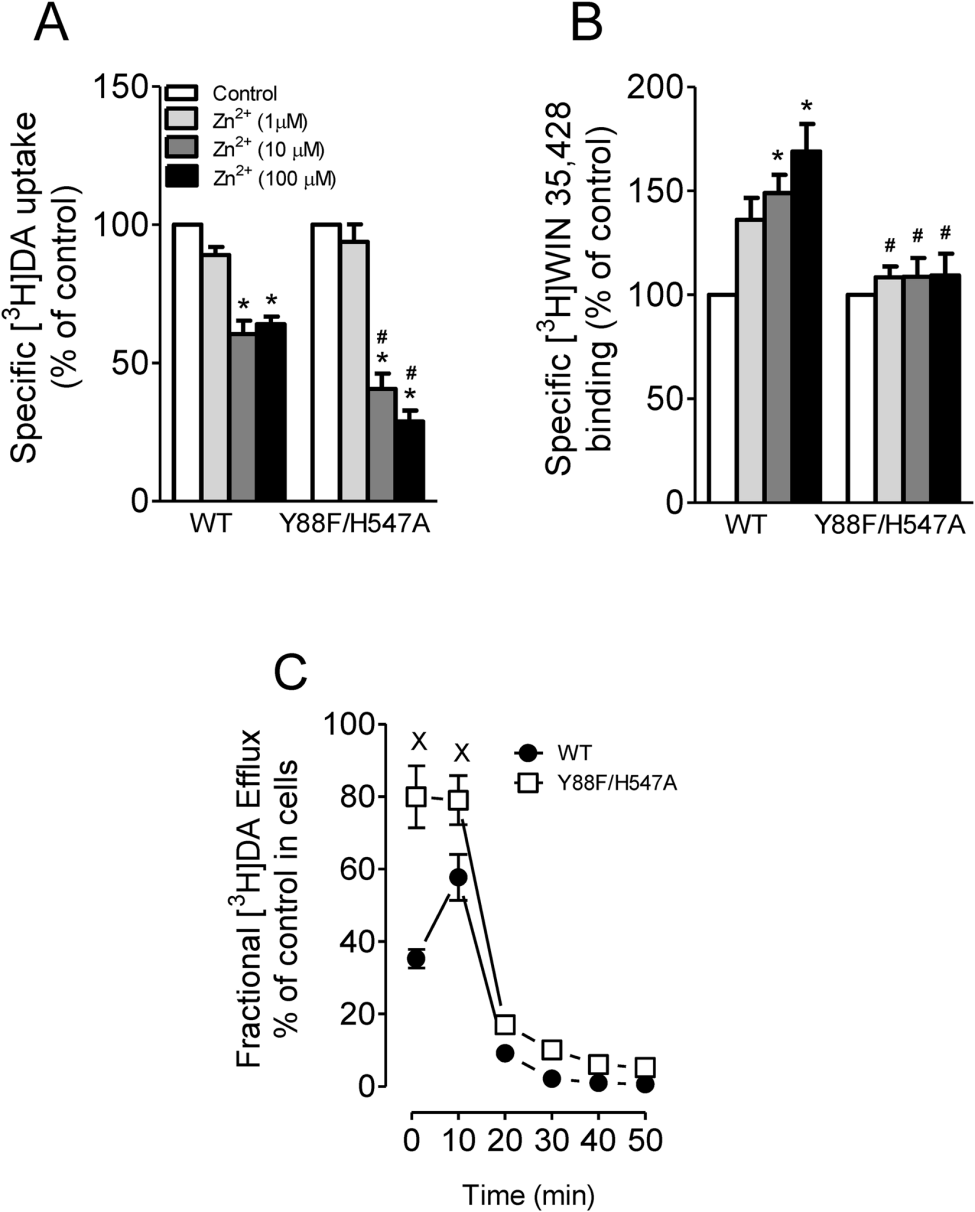
Effects of Y88F/H547A mutant on transporter conformational transitions. (**A**) [^3^H]DA uptake and (**B**) [^3^H]WIN 35,428 binding were conducted in PC12 cells transfected with WT hDAT or Y88F/H547A which were incubated with one of the concentrations of ZnCl_2_ (1, 10, 100 µM, final concentration) or KRH buffer (control) followed by addition of a fixed concentration of [^3^H]DA uptake or [^3^H]WIN 35,428 binding. The histogram shows [^3^H]DA uptake and [^3^H]WIN 35,428 binding expressed as mean ± S.E.M. of the respective controls set as 100% for individual experiment. *p < 0.05 compared to the respective controls (Bonferroni *t*-test) in [^3^H]DA uptake (n = 7/group) and [^3^H]WIN 35,428 binding (n = 9/group). ^#^*p* < 0.05 compared to WT hDAT with same concentration of ZnCl_2_ application (unpaired student *t*-test). (**C**) DAT-mediated basal DA efflux properties of WT hDAT and mutant. Functional DA efflux was conducted in PC12 cells transfected WT hDAT or Y88F/H547A by preloading with a fixed concentration of [^3^H]DA (0.05 μM, final concentration) at room temperature for 20 min followed by replacing with fresh buffer at indicated time points. Finally, the buffer in each well was separated from cells, and radioactivity in the buffer and remaining in the cells was counted. Each fractional efflux value of [^3^H]DA in WT hDAT and Y88F/H547A was expressed as percentage of total [^3^H]DA in the cells at the start of the experiment. Fractional efflux values of [^3^H]DA at 1, 10, 20, 30 and 40 min are expressed as the percentage of total [^3^H]DA with preloading with 0.05 μM (WT hDAT: 11044 ± 241 dpm and Y88F/H547A: 660 ± 123 dpm) present in the cells at the start of the experiment (n = 5/group). ^×^*p* < 0.05 compared to WT hDAT (Bonferroni *t*-test).

Tat protein has been shown to regulate DA transport allosterically, which may lead to alteration of transporter conformational transitions^19,22^. We have previously demonstrated that His547 residue plays a critical role in allosteric modulation of Tat-DAT interaction^18^. To determine whether the His547 residue still acts as a potential site for the regulation of Tat-DAT in Y88F/H547A, we examined the effects of Y88F/H547A on zinc modulation of [^3^H]DA uptake and [^3^H]WIN35,428 binding. Addition of Zn^2+^ is able to partially reverse an inward-facing state to an outward-facing state^31,32^. On the basis of this principle, the addition of Zn^2+^ to WT hDAT would inhibit DA uptake, whereas in a functional mutation of DAT, Zn^2+^ might diminish the preference for the inward-facing conformation and thus enhance DA uptake. As shown in Fig. 5A, two-way ANOVA analysis on the specific [^3^H]DA uptake in WT hDAT and Y88F/H547A revealed significant main effects of mutation [F_(1, 12)_ = 55.34, *p* < 0.0001], zinc [F_(3, 36)_ = 39.15, *p* < 0.0001], and mutation × zinc interaction [F_(3, 36)_ = 3.70, *p* < 0.05]. The addition of zinc significantly decreased the [^3^H]DA uptake in WT hDAT and its mutant in a concentration dependent manner. Compared to the respective controls (in absence of Zn^2+^), the addition of Zn^2+^ (10 and 100 µM) produced a similar reduction of [^3^H]DA uptake in WT (~40%, *ps* < 0.01) but a greater reductions (60% and 71% at 10 and 100 µM, respectively) in Y88F/H547A (*ps* < 0.01). This suggests that the double mutation of Y88/H547 does not affect zinc-induced regulation of DA uptake. In contrast, as shown in Fig. 5B, two-way ANOVA analysis of the specific [^3^H]WIN35,428 binding revealed significant main effects of mutation [F_(1, 16)_ = 5.82, *p* < 0.05], zinc [F_(3, 48)_ = 13.50, *p* < 0.0001], and mutation × zinc interaction [F_(3, 48)_ = 9.94, *p* < 0.0001]. Compared to the respective controls (in absence of Zn^2+^), addition of Zn^2+^ (10 and 100 µM) significantly increased [^3^H] WIN35,428 binding in WT hDAT (49% at 10 µM and 69% at 100 µM, *ps* < 0.05, Bonferroni *t*-test). The Zn^2+^ (10 and 100 µM)-induced increase in [^3^H]WIN 35,428 binding in WT hDAT was significantly diminished in Y88F/H547A [1 µM, *t*_(16)_ = 2.37, *p* < 0.05; 10 µM, *t*_(16)_ = 3.23, *p* < 0.01; 100 µM, *t*_(16)_ = 3.57, *p* < 0.01], suggesting that Y88F/H547A attenuates zinc-induced increase in [^3^H]WIN35,428 of hDAT by altering transporter conformational transitions.

To further determine the effect of Tyr88/His547 mutation on transporter conformational transitions, we examined basal efflux levels of [^3^H]DA in PC12 cells expressing WT hDAT and Y88F/H547A (Fig. 5C). twenty min after preloading with 0.05 µM [^3^H]DA, cells were washed and fractional DA efflux samples were collected at the indicated time. Two-way ANOVA revealed significant main effects of mutation [F_(1, 8)_ = 59.38, *p* < 0.0001], time [F_(5, 40)_ = 116.35, *p* < 0.0001], and mutation × time interaction [F_(5, 40)_ = 8.16, *p* < 0.0001]. Post hoc analyses showed that compared to WT hDAT, DA efflux levels were significantly elevated at 1 and 10 min (*ps* < 0.05, Bonferroni *t* test).

### Effects of Y88/H547 mutation on amphetamine-regulated DA efflux and uptake

**Figure 6:**
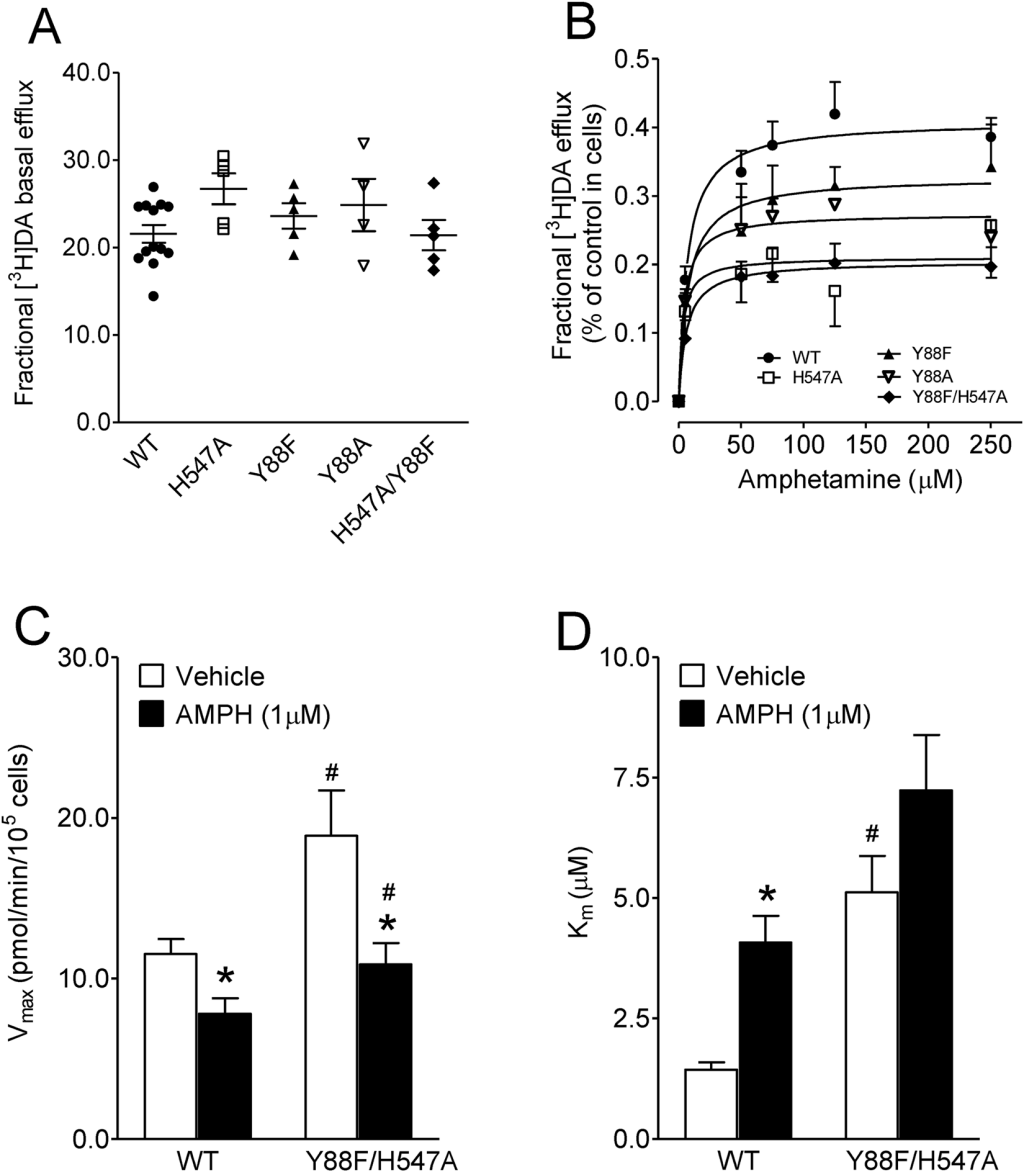
Effects of AMPH on the kinetic analysis of DA efflux and uptake. PC12 cells transfected with WT hDAT and its mutants were washed, and preloaded with KRH buffer containing 50 nM [^3^H]DA for 20 min at room temperature. After three quick washes with KRH, aliquots from each well were collected and replaced with fresh KRH every 5 min for 15 min to establish basal efflux. At 20 min, AMPH (5–250 µM) was added, and the same aliquots were taken after 5 min as described in *Material and Methods*. (**A**) Baseline of [^3^H]DA efflux before addition of AMPH. Data were presented as means ± S.E.M. of the ratios of efflux to [^3^H]DA in cells (efflux dmp/cellular dmp) in WT hDAT and mutants (n = 4–13). (**B**) AMPH-mediated efflux is expressed as means ± S.E.M. of the fractional release of cellular [^3^H]DA per 5 min in WT and mutants. (**C,D**) kinetic analysis of [^3^H]DA uptake in WT hDAT and Y88F/H547A in the presence or absence of AMPH (1 µM). Data were presented as means ± S.E.M. (n = 5/group). *p < 0.05 compared to their respective controls (paired *t* test) ^#^*p* < 0.05 compared to WT hDAT value (unpaired Student’s *t* test).

AMPH acts as a substrate for DAT which subsequently reverses the transporter leading to DA efflux^33,34^. AMPH-stimulated [^3^H] DA efflux in PC12 cells transfected with WT hDAT and its mutants was determined under a stable baseline of DA efflux (Fig. 6A), showing no difference on basal [^3^H]DA efflux in control groups (F_(4, 27)_ = 1.92, *p* = 0.14, one-way ANOVA). As shown in Fig. 6B, AMPH induced [^3^H]DA efflux in WT hDAT and mutants in a concentration dependent manner. Two-way ANOVA analysis on the V_max_ values revealed significant main effects of mutation [F_(4, 26)_ = 4.63, *p* < 0.01], AMPH [F_(5, 130)_ = 76.81, *p* < 0.0001] and mutation × AMPH interaction [F_(20, 130)_ = 2.34, *p* < 0.01]. As shown in Table 3, the V_max_ values of [^3^H]DA efflux in response to AMPH were reduced in H547A (0.22 ± 0.04) and Y88F/H547A (0.20 ± 0.03) compared to WT hDAT (0.42 ± 0.03, *ps* < 0.05, Bonferroni’s multiple comparison test). No difference in K_m_ was found among WT hDAT and all mutants.

**Table 3:** Kinetic analysis of AMPH-stimulated [^3^H]DA efflux in WT hDAT and mutants.

	WT hDAT	Y88F	Y88A	H547A	Y88F/H547A
*V*_max_	0.42 ± 0.03	0.28 ± 0.06	0.29 ± 0.01	0.22 ± 0.04**	0.20 ± 0.03**
*K*_m_	10.2 ± 3.2	7.61 ± 2.00	9.80 ± 5.11	9.49 ± 6.52	9.31 ± 4.7

Data are presented as mean ± S.E.M. of the *V*_*max*_ and *K*_*m*_ values for AMPH-stimulated [^3^H]DA in WT hDAT and its mutants from 5–13 independent experiments. *p < 0.05 compared to WT hDAT values (Bonferroni’s multiple comparison test).

Double mutation of Tyr88 and His547 could induce DAT conformational change by altering AMPH interaction with DAT. We first examined the affinity for AMPH to inhibit [^3^H]DA uptake in WT and Y88F/H547A (Table 1), however, no significant difference in the IC_50_ value was found between WT (926 ± 167.4 nM) and Y88F/H547A [1279 ± 200.5 nM, *t*_(8)_ = 1.32, *p* = 0.22]. Next, we performed the kinetic analysis of [^3^H]DA uptake in WT and Y88F/H547A in presence of AMPH (1 µM, final concentration). As shown in Fig. 6C, two-way ANOVA analysis on the V_max_ of [^3^H]DA uptake reveled significant main effects of mutation (F_(1, 16)_ = 9.50, *p* < 0.01) and AMPH (F_(1, 16)_ = 11.99, *p* < 0.01). No significant interaction of mutation × AMPH treatment was found (F_(1, 16)_ = 1.61, *p* = 0.22). Post hoc analyses show that the V_max_ value in Y88F/H547A (18.9 ± 2.81 pmol/min/10^5^ cells) was higher than that in WT hDAT (11.5 ± 0.94 pmol/min/10^5^ cells, *t*_(8)_ = 2.49, *p* < 0.05, n = 5/group) in the absence of AMPH. AMPH decreased the V_max_ in both WT hDAT (32%, 7.80 ± 0.97 pmol/min/10^5^ cells, *t*_(8)_ = 2.76, *p* < 0.01) and Y88F/H547A (42%, 10.9 ± 1.33 pmol/min/10^5^ cells, *t*_(8)_ = 2.58, *p* < 0.05) compared with their respective controls. There was a significant difference in the magnitude of the AMPH-induced decrease in V_max_ values between WT hDAT and Y88F/H547A (*t*_(8)_ = 1.86, *p* < 0.05). Furthermore, two-way ANOVA analysis on K_m_ values revealed significant main effects of mutation [F_(1, 16)_ = 21.06, *p* < 0.001] and AMPH [F_(1, 16)_ = 10.17, *p* < 0.01],whereas no significant interaction of mutation × AMPH treatment was found [F_(1, 16)_ = 0.13, *p* = 0.73]. In the absence of AMPH, the K_m_ values of [^3^H]DA uptake was greater in Y88F/H547A (5.12 ± 0.76 µM) than that in WT hDAT [1.43 ± 0.15 µM, *t*_(8)_ = 4.79, *p* < 0.01). Compared to the controls, AMPH significantly increased the K_m_ value in WT hDAT [4.08 ± 0.56 µM; *t*_(8)_ = 4.63, *p* < 0.01], which was attenuated in Y88F/H547A (7.24 ± 1.15 µM).

## Discussion

Our recent studies demonstrated that single hDAT mutants, Y88F, K92M, and H547A differentially regulate DAT reuptake but attenuate Tat’s inhibitory effect on DAT function. This study sought to determine whether multiple mutations on these hDAT residues (Y88F/H547A) and (Y88F/K92M/H547A) influence the direct interactions between Tat and DAT. There were three major findings. First, compared with WT hDAT, Y88F/H547A enhances V_max_ of DA uptake, whereas Y88F/K92M/H547A dramatically reduces the V_max_. Second, consistent with the computational modeling prediction, Y88F/H547A attenuates Tat-induced inhibition of DA uptake observed in WT hDAT. The third finding is that Y88F/H547A displays an attenuation of zinc ion modulation of [^3^H]WIN35,428 binding, increased basal DAT-mediated DA efflux, and reduced AMPH-stimulated DA efflux. Collectively, these findings suggest that targeting DAT residues Tyr88 and His547 may have potential therapeutic benefits on Tat-induced dysregulation of dopaminergic system seen in patients with HAND.

According to our computationally modeled structures, Y548-Y470-Y551 is a stable motif (denoted as the YYY motif for convenience) essential for the conformational conversion of hDAT required for the DA transport process^35^. This YYY motif has hydrophobic contact with a typical U-turn loop associated with extracellular loop 6 (EL6) and transmembrane 10 (TM10a). Any structural changes affecting the stability of the YYY motif could destabilize the R85-D476 salt bridge of hDAT, and the salt bridge is key for the conformational conversion^36–39^. H547 is expected to significantly affect the hDAT stability because H547 is adjacent to Y548 of the YYY motif^35^, which is consistent with the observed increase in DA uptake by the H547A mutation^18^. On the other hand, the side chain of K92 residue (TM1b) forms a favorable salt bridge with the side chain of D313 (TM6a) during the molecular dynamics simulation on the hDAT-DA binding complex, and the bridge is also essential for the conformational conversion of hDAT required for the DA uptake process^40^. For this reason, disrupting the K92-D313 salt bridge will likely impair or slow down the DA uptake process, which has been validated pharmacologically by mutations on K92 (K92M)^17^ and D313 (D313N)^41^. In addition, the aromatic ring on the side chain of Y88 is sandwiched by TM1b and extracellular loop 4 (EL4) in a hydrophobic region of hDAT. Therefore, mutation of Y88 to phenylalanine retains the normal reuptake function of hDAT and demonstrated no difference compared to WT hDAT^17^. However, Y88F increased the K_m_ of DA binding by about 50%, suggesting that Y88F may slightly alter the local structure around Y88^17^. In the current study, the double mutant Y88F/H547A retains the increased V_max_ observed in H547A, suggesting a critical role of the mutated H547 residue in regulating transporter activity. In contrast, the combination of the mutated residues Y88, K92, and H547 does not follow a simple math addition, because compared to WT hDAT, a 96% reduction of V_max_ was observed in Y88F/K92M/H547A, whereas the V_max_ was decreased by 71% in K92M^17^. These results suggest that the inclusion of the K92 mutation in the K92-D313 salt bridge is a critical factor in the differential DAT kinetics in the triple mutation of hDAT. As Y88 is spatially adjacent to R85 and K92 in helix TM1b, the double mutations on both Y88F and K92M are expected to induce more structural disturbance to the R85-D476 and K92-D313 salt brides, thus further decreasing V_max_ of the transporter. Therefore, triple mutant Y88F/K92M/H547A has a lower V_max_ compared to, single mutant K92M. Although including K92M mutation significantly decreases the V_max_ for DA uptake in the triple mutant Y88F/K92M/H547A compared to Y88F/H547A, no significant change in the B_max_ of [^3^H]WIN 35,428 binding is found in the double or triple mutants, suggesting that the multiple mutations may alter DAT re-uptake function rather than the substrate binding sites on DAT. This conclusion is also supported by the increased K_m_ in Y88F/H547A and no change in K_m_ in Y88F/K92M/H547A. However, single point mutations of Y88, K92, and H547 differentially affect the V_max_ with no changes in the K_m_^17,18^. One possible explanation is that allosteric modulation of the dynamic DA transport process may be responsible for the enhanced V_max_ and its associated DA uptake potency.

We further determined the effect of double mutant Y88F/H547A on the basal DA uptake via hDAT and binding potency for DA, GBR12909, and cocaine. Y88F/H547A had decreased the potency for [^3^H]DA to inhibit DA uptake relative to WT hDAT, which is similar to that in Y88F and H547A^16,18^. However, Y88F/H547A increased the DA uptake potency for cocaine and GBR12909, which is consistent with the single mutation of Y88F relative to WT hDAT^17^. Similar to Y88F^17^, Y88F/H547A displayed decreased potency for DA to inhibit [^3^H]WIN35,428 binding. However, the inhibition potency of [^3^H]WIN35,428 binding for cocaine was decreased in Y88F/H547A but increased in Y88F^17^. Y88F/H547A, similar to Y88F^17^, retained the [^3^H]WIN35,428 binding potency for GBR12909. GBR12909 is a selective inhibitor of DA uptake, binding to the piperazine acceptor site without influencing DA release^42^; while cocaine is a competitive inhibitor for DAT and prefers to an outward-facing transport state, leading to a decrease in DA transport^43,44^. According to our computational modeling prediction, both hDAT residues Y88 and H547 are the critical binding sites on DAT for Tat^20^. Therefore, our findings demonstrate that through allosteric modulation, mutation of Y88 and H547 alters the inhibition potency for DA uptake and DAT binding site for substrate and DAT inhibitors^40^.

Our previous study has demonstrated that mutant H547A displays increased sensitivity to PKC-mediated downregulation of DAT function by PMA compared to WT hDAT^18^. Interestingly, the current study shows no difference in PMA-mediated reduction of DA uptake between Y88F/H547A and WT hDAT, indicating that Y88F/H547A-induced increase in V_max_ is independent of PKC activity. It has been shown that PKC-mediated phosphorylation of DAT downregulates DA uptake velocity^45–47^. Through allosteric modulatory effect, single mutation of H547 may alter the levels of PKC-induced phosphorylation of DAT activity, thereby causing the increased V_max_. However, double mutation of Y88 and H547 may attenuate the intermolecular structural interaction across PKC phosphorylation sites. Indeed, compared with WT hDAT, H547A, and Y88F/H547A enhances the V_max_ values by 196%^18^ and 96%, respectively. In addition to PKC-mediated regulation of DAT function, activation of palmitoyl acyltransferase, a group of enzymes that transfer palmityl group to -SH group on cysteine on a protein, enhances DA uptake via DAT palmitoylation^48^. Therefore, we will investigate whether Y88F/H547A-induced increased DA uptake is dependent on palmitoylation activity.

A major finding is that the double mutant Y88F/H547A completely attenuates the Tat-mediated inhibition of DAT reuptake function, which is consistent with our previous reports on Y88F- or H547A-induced attenuation of the Tat’s effect^17,18^. According to our computational modeling prediction, either the Y88 or H547 residue could form hydrogen bonds with Tat residue K19 or R49 independently, suggesting that the double mutation of Y88 and H547 eliminated Tat-DAT binding. Hence, our pharmacological study further validates the computational prediction for the complementary hydrophobic interactions between Tat and hDAT. Furthermore, since we have demonstrated that Tat interacts with DAT allosterically^19,22^, the current findings also suggest that double mutation of Y88 and H547 attenuates Tat effect through a conformational mechanism. First, Y88F/H547A attenuates zinc-mediated increase in [^3^H]WIN35,428 binding relative to WT hDAT. Second, Y88F/H547A augments basal DA efflux when compared to WT hDAT, which opposes our previous reports showing that neither Y88F nor H547A affected the basal efflux^17,18^. The elevated basal DA efflux in Y88F/H547A may be attributed to its elevated V_max_ of DA uptake and DA accumulation. Taken together, Y88F/H547A, via an alteration of DAT conformational transitions, may influence the binding structure of hDAT-Tat complex, thereby disrupting Tat-induced inhibition of DA uptake. Studying the multiple mutation of DAT residues in Tat-DAT intermolecular interaction will greatly contribute to our ongoing proof of concept studies using novel allosteric modulators to establish their potential for therapeutic application in HAND.

AMPH, a substrate of DAT, competitively inhibits the reuptake of DA and elicits DAT-mediated DA efflux by reversal of the transporter^49^. Our computational modeling has demonstrated that the dynamic DA uptake process includes outward-open, outward-occluded, and inward-open states^21^, which can be reversed by DA substrate and DAT mutation^49–51^. The current results indicate that the potency for AMPH inhibition of [^3^H]DA uptake was not altered in Y88F/H547A compared with WT hDAT, suggesting that the mutation of Tyr88 and His547 does not alter the interaction of AMPH with hDAT. However, AMPH-mediated DA efflux was reduced in H547A and Y88F/H547A, suggesting that mutations of Tyr88 and His547 residues alter the conformational equilibrium of the DAT transport transitions. Furthermore, AMPH decreases the V_max_ of DA uptake in both WT hDAT and Y88F/H547A, which is consistent with the previous report^51^, however, the magnitude of the AMPH-mediated reduction of the V_max_ in Y88F/H547A was greater than that in WT hDAT. Interestingly, the AMPH-mediated reduction of the V_max_ was accompanied by increased K_m_ values in WT hDAT but not Y88F/H547A. Given that DAT-mediated DA transport is accompanied by reuptake of two Na^+^ and Cl^−^ ions, AMPH influx would transport Na^+^ through DAT and enhance its levels at the inner face of the transporter^52^. Therefore, AMPH decreases DA transport but increases K_m_ values in WT hDAT, whereas altered transporter conformation by Y88F/H547A may attenuate the increased K_m_. Taken together, the results along with attenuated zinc-regulated [^3^H] WIN35,428 binding and increased basal DA efflux in Y88F/H547A further support that mutations of Tyr88 and His547 cause an alteration in conformational transitions, thereby disrupting the inhibitory effect of Tat on DAT reuptake function.

In conclusion, our data further demonstrate DAT Tyr88 and His547 as important residues for DAT function and the Tat-DAT interaction. Double mutation of these residues preserves single mutant H547A-mediated enhancement of DA uptake and diminishes Tat-induced inhibition of DA transport^18^. An allosteric modulatory mechanism may contribute to Y88F/H547A-mediated attenuation of Tat-induced inhibition of DA transport. Understanding the multiple mutation effects of the identified DAT residues might provide a basis for a novel approach for developing compounds to attenuate Tat binding to DAT by an allosteric mechanism in HIV-1-infected individuals.

## Materials and Methods

### Prediction of Tat-DAT binding model

We performed computational modeling and simulations to model the binding structure of hDAT with HIV-1 clade B type Tat based on the nuclear magnetic resonance (NMR) structures of Tat^53^ and the constructed structure of hDAT-DA complex^21^. Protein docking and molecular dynamics simulations were employed to identify the conformation of hDAT-Tat complex. The energy-minimized complex structure used in this work were extracted from long-time equilibrated molecular dynamics simulation trajectories in our previous work^17,18,20,35^.

### Construction of plasmids

We selected the mutations of Y88, H547, Y88/H547 and Y88/K92/H547 based on predictions of computational modeling and simulations (Fig. 1) and our previous studies^17,18^. Single mutants, Y88F, K92M, and H547A, are expected to abolish hydrogen bonds between hDAT and Tat. Double (H547A/Y88F) and triple (H547A/Y88F/K92M) mutations were generated based on the wild type hDAT (WT hDAT) sequence (NCBI, cDNA clone MGC: 164608 IMAGE: 40146999) by site-directed mutagenesis. We then used the synthetic cDNA encoding hDAT subcloned into pcDNA3.1 + as a template to generate mutants using QuikChange™ site-directed mutagenesis Kit (Agilent Tech, Santa Clara CA). We performed the DAT sequence for confirming the sequence of the mutant construct at University of South Carolina EnGenCore facility. Plasmids DNA were propagated and purified using a plasmid isolation kit (Qiagen, Valencia, CA, USA).

### Cell culture and DNA transfection

In the current study, we used rat pheochromocytoma cells (PC12 cells, CRL-1721, American Type Culture Collection (ATCC), Manassas, VA), which were maintained in Dulbecco’s modified eagle medium (DMEM, Life Technologies, Carlsbad, CA) supplemented with 15% horse serum, 2.5% bovine calf serum, 2 mM glutamine and antibiotics, 100 U/mL penicillin and 100 µg/mL streptomycin. Cells were cultured at 37 °C in a 5% CO_2_ incubator. Once cells were growing to 100% confluence on plates, cells were seeded at a density of 1 × 10^5^ cells/cm^2^ in 24-well plates and transfected with WT hDAT or mutants by Lipofectamine 2000 (Life Technologies). Twenty-four hours after transfection, intact cells or cell suspensions were used for experiments.

### [^3^H]DA uptake assay

In order to determine whether DAT mutants alter DAT function, we evaluated the maximal velocity (V_max_) or Michaelis-Menten constant (K_m_) of [^3^H]DA uptake in intact PC12 cells transfected with WT hDAT or mutants as reported previously^16,17^. Twenty-four hours after transfection, cells in 24-well plates were washed twice with Krebs-Ringer-HEPES (KRH) buffer (final concentration in mM: 125 NaCl, 5 KCl, 1.5 MgSO_4_, 1.25 CaCl_2_, 1.5 KH_2_PO_4_, 10 D-glucose, 25 HEPES, 0.1 EDTA, 0.1 pargyline, and 0.1 L-ascorbic acid; pH 7.4) and incubated with nomifensine (10 µM, final concentration) as nonspecific uptake for 10 min at room temperature. A fixed concentration of [^3^H]DA (500,000 dpm/well, specific activity, 21.2 Ci/mM; PerkinElmer Life and Analytical Sciences, Boston, MA) mixed with one of six concentrations of unlabeled DA (final DA concentrations, 0.03–5 µM) were applied to cells at room temperature for additional 8 min. The specific DAT-mediated DA uptake was calculated by subtracting non-specific uptake from total uptake (in the absence of nomifensine). The reaction was terminated by removal of solution from wells and quickly washed three times with ice cold KRH buffer. Cells were then lysed in 500 μL of 1% SDS for an hour and radioactivity was measured using a liquid scintillation counter (Tri-Carb 2900TR; PerkinElmer Life and Analytical Sciences, Waltham, MA).

To confirm whether Y88F/H547A-induced increase in V_max_ was dependent on PKC regulatory mechanism, the V_max_ of [^3^H]DA uptake was measured in the presence or absence of 1 µM PMA, a PKC activator (Tocris, Bristol, UK) as the previous reports^29,30^. Intact PC12 cells transfected with WT or Y88F/H547A were preincubated with or without PMA for 30 min at 37 °C followed by additional 8 min incubation with six concentrations of mixed [^3^H]DA as described above. To determine the effect of amphetamine (AMPH) on Y88F/H547A-mediated transporter conformational transitions, V_max_ or K_m_ of [^3^H]DA uptake in WT and Y88F/H547A was determined in the presence or absence of 1 µM AMPH (Sigma-Aldrich, St. Louis, MO) as reported previously^51^.

To determine whether these mutations alter the affinity of DAT substrate or inhibitors as well as Zn^2+^-regulation of DA transport, we performed the competitive inhibition of DA uptake in intact PC12 cells transfected with WT or mutant, which were preincubated with a series of final concentrations of DA (1 nM-1 mM), GBR12909 (1 nM-10 µM), cocaine (1 nM-1 mM), AMPH (1 nM-1 mM) or ZnCl_2_ (1, 10, and 100 μM) at room temperature for 10 min followed by additional 8 min incubation with a fixed concentration of [^3^H]DA (0.05 µM, final concentration).

In order to determine whether double mutation of Y88 and H547 disrupts Tat and DAT interaction, we performed the specific [^3^H]DA uptake in PC12 cells transfected with WT or mutant in the presence or absence of Tat protein. In brief, cells were dissociated with trypsin/EDTA (0.25%/0.1%, 1 mL for one 10 cm dish) and resuspended in culture medium and incubated at room temperature for 10 min. The dissociated cells were collected by centrifugation at 400 × g for 5 min at 4 °C and washed once with phosphate-buffered saline followed by additional 5 min centrifugation (400 × g, 4 °C). Finally, the resulted cell pellets were resuspended in KRH assay buffer. The cell suspensions from WT hDAT or Y88F/H547A mutant were then preincubated with or without recombinant Tat_1–86_ (rTat_1–86_, 140 nM, final concentration, Diatheva, Fano, Italy) at room temperature for 20 min followed by additional 8 min incubation with a mixed [^3^H]DA uptake (0.05 µM, final concentration). Non-specific [^3^H]DA uptake was determined in the presence of 10 µM nomifensine. The update reaction was terminated by immediate filtration through Whatman GF/B glass filters (presoaked with 1 mM pyrocatechol for 3 h) followed by three washes with 3 mL of ice-cold KRH buffer containing pyrocatechol using a Brandel cell harvester (model M-48; Brandel Inc., Gaithersburg, MD). Radioactivity was determined as described above.

### [^3^H]WIN 35,428 Binding Assay

Binding assays were used to determine the kinetic parameters (B_max_ or K_d_) of [^3^H]WIN 35,428 binding in intact PC12 cells transfected with WT hDAT or mutant. Cells were washed with sucrose-phosphate buffer twice (final concentration in mM: 2.1 NaH_2_PO_4_, 7.3 Na_2_HPO_4_7H_2_O, and 320 sucrose, pH 7.4) and then incubated with one of the six concentrations of [^3^H]WIN 35,428 (84 Ci/mmol, PerkinElmer, 0.5–30 nM final concentrations) in a final volume of 500 μL on ice for 2 h. Nonspecific binding at each concentration of [^3^H]WIN 35,428 was determined by adding in the presence of 30 µM cocaine (final concentration) and subtracted from total binding to obtain the specific binding. For competitive inhibition experiments, assays were performed in a final volume of 250 μL. Intact PC12 cells transfected with WT or mutated hDATs were incubated in buffer containing 25 μL of [^3^H]WIN 35,428 (final concentration, 5 nM) and one of ten concentrations of DA (1 nM- 100 μM), cocaine (1 nM- 100 μM), GBR12909 (0.01 nM- 1 μM) or ZnCl_2_ (1, 10, 100 μM) on ice for 2 h. Assays were terminated by removing reaction reagents in each well followed by two washes with ice-cold assay buffer. Cells were lysed with 1% SDS for an hour and subjected to the radioactivity measurement as described above.

### [^3^H]DA efflux assay

Basal DA efflux was performed at room temperature as described previously^16–18^. Intact PC12 cells transfected with WT hDAT or its mutant were preloaded with 0.05 μM [^3^H]DA for 20 min and then washed 3 times with KRH buffer prior to collecting fractional efflux samples. To obtain an estimate of the total amount of [^3^H]DA in the cells at the zero time point, cells from a set of wells (four wells/sample) were lysed rapidly in 1% SDS after preloading with [^3^H]DA. To collect factional efflux samples, buffer (500 μL) was added into a separate set of cell wells and transferred to scintillation vials after 1 min as an initial fractional efflux, and another 500 μl buffer was added to the same wells and collected after 10 min as second fractional efflux. Additional fractional efflux at 20, 30, 40, 50 min, respectively, was repeated under the same procedure. After last fractional efflux, cells were lysed and counted as total amount of [^3^H]DA remaining in the cells from each well.

To further determine DAT mutation-mediated transporter confirmation transitions, basal efflux and AMPH-stimulated DA efflux in WT hDAT and its mutants were measured as reported previously^51^. PC12 cells transfected with WT hDAT and its mutants growing in 24-well plates were preloaded with [^3^H]DA (50 nM, final concentration) for 20 min at room temperature. Subsequently, cells in all plate wells were washed with KRH for three times, cells were then incubated with KRH buffer containing AMPH (5–250 µM) or vehicle (KRH buffer alone, as baseline control) for 15 min at room temperature. After incubation, the buffer solution incubated with AMPH or vehicle was collected as AMPH-stimulated efflux or baseline efflux. Cells were lysed in 1% SDS and subject to count for obtaining the initial amount of DA (total DA) in the cells.

### Data analysis

In the current study, all data are presented as mean ± SEM with *n* as the number of independent experiments for each experiment group. Pharmacological parameter values (V_max_, K_m_, B_max_, and K_d_) and IC_50_ from [^3^H]DA uptake and [^3^H]WIN 35,428 binding were calculated by nonlinear regression analysis using a one-site model with variable slope. To determine the significant difference in the pharmacological parameters between unpaired sample groups, such as WT hDAT and its mutants, unpaired Student’s *t* test was used for the statistical comparisons. Significant differences between sample groups were analyzed with separate ANOVAs followed by post-hoc, Bonferroni multiple comparison test as indicated in the results Section of each experiment. All statistical analyses were performed using IBM SPSS Statistics version 25, and differences were considered significant at *p* < 0.05.
